# Depression in patients with spondyloarthritis: prevalence, incidence, risk factors, mechanisms and management

**DOI:** 10.1177/1759720X20970028

**Published:** 2020-11-02

**Authors:** Joel T. Parkinson, Éimear M. Foley, Deepak R. Jadon, Golam M. Khandaker

**Affiliations:** Department of Psychiatry, School of Clinical Medicine, University of Cambridge, Herchel Smith Building for Brain and Mind Sciences, Cambridge Biomedical Campus, Robinson Way, Cambridge, Cambridgeshire CB2 0SZ, UK; Department of Psychiatry, School of Clinical Medicine, University of Cambridge, Cambridge, UK; Department of Rheumatology, Cambridge University Hospitals NHS Foundation Trust, Cambridge, UK; Department of Medicine, University of Cambridge, UK; Department of Psychiatry, School of Clinical Medicine, University of Cambridge, Cambridge, UK; Cambridgeshire and Peterborough NHS Foundation Trust, Cambridge, UK

**Keywords:** ankylosing spondylitis, assessment, depression, depressive disorder, epidemiology, psoriatic arthritis, spondyloarthritis, treatment

## Abstract

Depression is a major neuropsychiatric disorder common in patients with rheumatological conditions including spondyloarthritis (SpA). It is associated with higher disease activity, functional impairment, poor treatment response and quality of life in patients with musculoskeletal disorders. Using ankylosing spondylitis (AS) and psoriatic arthritis (PsA) as examples, we have reviewed the evidence regarding the burden, risk factors, potential mechanisms and clinical management of depression in spondyloarthritis. The prevalence of depression is higher in patients with AS and PsA compared with the general population, with evidence of moderate/severe depression in about 15% of patients with AS or PsA. Mild depression is even more common and estimated to be present in about 40% of patients with AS. In addition to conventional risk factors such as stressful life events and socioeconomic deprivation, increased risk of depression in SpA may be associated with disease-related factors, such as disease activity, poor quality of life, fatigue, and sleep disturbances. Emerging evidence implicates inflammation in the aetiology of depression, which could also be a shared mechanism for depression and chronic inflammatory conditions such as AS and PsA. It is imperative for clinicians to actively assess and treat depression in SpA, as this could improve treatment adherence, quality of life, and overall long-term clinical and occupational outcomes. The use of validated tools can aid recognition and management of depression in rheumatology clinics. Management of depression in SpA, especially when to refer to specialist mental health services, are discussed.

## Introduction

Spondyloarthritis (SpA) is a group of chronic auto-inflammatory diseases characterised by peripheral and/or axial inflammation. Ankylosing spondylitis (AS) is characterised by axial inflammation and is the archetypal and most common type of spondyloarthritis, with regional prevalence ranging from 0.02–0.35%.^1^ Psoriatic arthritis (PsA), on the other hand, is characterised predominantly by peripheral inflammation. Prevalence of PsA remains largely unknown due to lack of data from some regions and due to differing classifications, but current estimates are about 0.02–0.25%.^2^ Though distinct, both diseases share common pathological features, with inflammation playing a crucial role. For instance, HLA-B27 gene polymorphisms are implicated in various spondyloarthritis.^3^ HLA-B27 is associated with spinal involvement,^4^ which may explain its strong association with AS,^5^ where it accounts for about 30% of heritability of the illness.^6^ Similarly, dysregulation of interleukin 17/23 pathway is implicated in the pathogenesis of both AS and PsA.^7,8^ Other implicated inflammatory cytokines include tumour necrosis factor alpha (TNF-α), IL-6 and IL-22.^9,10^ Such pathophysiologic similarity is reflected in common clinical features. For example, psychiatric symptoms/disorders, especially depression, are common in both AS and PsA, which may be linked with potential shared mechanisms such as inflammation.

Depression is a common and serious mental disorder that affects about 10–20% of the population in the lifetime,^11^ with a quarter of these cases beginning before age 20 years.^12^ It is one of the leading contributors of disability-adjusted life-years globally.^13^ Depressive symptoms can be broadly grouped as somatic or psychological. Somatic symptoms include fatigue, sleep disturbance, changes in appetite/weight, psychomotor agitation/retardation, and anhedonia (loss of pleasure). Psychological symptoms can include hopelessness, low self-esteem, excessive/inappropriate guilt and suicidality.^14^ Typically, depression has a relapsing remitting nature, with high recurrence rate; up to 50% after the first episode and up to 90% after the third episode.^15–17^ Similar to most chronic illnesses of adult life, depression is a multifactorial condition with contributions from both genetic and environmental factors. Recent genome-wide association studies have identified over 100 genetic variants associated with depression.^18–20^ These include genetic variants linked to specific brain regions such as the prefrontal cortex (SORCS3 on chromosome 10 and NEGR1 on chromosome 1),^19^ and neuro-transmission/signalling involving calcium (CACNA1E and CACNA2D1), dopamine (DRD2), and glutamate (GRIK5 and GRM5).^18,20^ The majority of genes identified were in the extended major histocompatibility complex (MHC) region,^20^ a region that is essential for autoimmunity.^21^ The cumulative effect of these variants on risk is relatively modest, with common single nucleotide polymorphisms (SNPs) contributing about 9% variation in depression liability.^18^ Notable environmental risk factors include stressful life events and early-life adversity such as abuse/maltreatment.

Pathophysiologic explanation and pharmacotherapy for depression is predicated on monoamine neurotransmitters, particularly serotonin. However, about a third of patients with depression do not respond to monoaminergic treatment such as selective serotonin reuptake inhibitors (SSRIs),^22^ suggesting other mechanisms contribute to illness risk. Emerging evidence indicates a potentially causal role for inflammation in the pathogenesis of depression.^23–25^ Depression is often comorbid with physical illnesses, notably cardiovascular disease and auto-immune/auto-inflammatory conditions such as arthritis.^26^ Inflammation could be a shared mechanism for depression and cardiovascular and possibly other comorbid physical illnesses such as type 2 diabetes.^22,27,28^

Depression is a clinically relevant issue for rheumatologists as depressive symptoms are present in over a third of patients with rheumatological conditions, including rheumatoid arthritis (RA), axial spondyloarthritis and PsA.^29–31^ Depression is associated with poor adherence to treatment for chronic physical illness.^32^ Persistent depressive symptoms have been reported to not only exacerbate disease activity and disability but also potentially reduce treatment response.^33,34^ As a result, treating depression alone can improve rheumatological outcomes.

In this review, we discuss the current literature regarding the burden of depression in spondyloarthritis using AS and PsA as examples. We discuss prevalence, incidence, risk factors and potential mechanisms for the higher risk of depression in patients with AS and PsA. We review the current guidance on the management of depression as relevant to rheumatology clinicians.

## Prevalence and incidence of depression in patients with AS or PsA

Depression is common in patients with rheumatological conditions including SpA with evidence for a complex bi-directional relationship. For instance, depression may exacerbate disease activity in patients with AS or PsA, while disease severity and certain symptoms can contribute to low mood. A Swedish population-based study reported that individuals with rheumatological disease had a higher risk of psychiatric disorders than the general population, with incidence rates showing an increase of 48.2 men per 100,000, and 45 women per 100,000.^35^ Those with AS or systemic lupus erythematosus (SLE) had a higher risk of subsequent psychiatric disorder(s) compared with patients with RA, while depression was the leading cause for psychiatric hospitalisation in patients with AS.^35^ However, this study examined severe psychiatric illness requiring hospitalisation as the outcome, and so may not reflect the burden of less severe psychiatric morbidity. A more recent study of comorbidities in axial spondyloarthritis (axSpA) used clinical information from clinical notes, and primary and secondary medical records to investigate a broader spectrum of comorbidities, not limited to hospitalisation or psychiatric illness. Depression was reported to be one of the most common comorbidities in axSpA, present in about 15% of patients. Using hierarchical clustering of 38 conditions in 419 patients with axSpA the study identified 15 clusters. The depression-anxiety group was one of the most prevalent clusters, with patients in this group scoring higher on disease activity and lower on quality of life measures.^36^

Three systematic literature reviews and meta-analyses on the prevalence of depression in AS and PsA are reported.^31,37,38^ The largest meta-analysis included 19,263 patients with AS from 33 studies conducted in Europe, Asia and South America. These studies measured depression using a variety of methods including self-report, validated questionnaires, clinical interviews, administrative and hospital records. In one meta-analysis, the pooled prevalence of depression in AS was reported to be 29%.^37^ However, findings from this meta-analysis were largely driven by one large study from Taiwan comprising 11,701 patients,^39^ which used the Elixhauser comorbidity index to record ICD-9 identified diagnoses of depression,^40^ rather than direct assessment of mood using a validated scale/interview. Reliance on clinical diagnosis can lack temporal validity, provides no information on current symptom severity, and may underestimate prevalence due to subthreshold and undiagnosed depression.

Although another meta-analysis comprised fewer studies, the assessment of depression was more robust due to the inclusion criteria that studies must use a validated diagnostic and screening criteria for depression, with a defined threshold.^31^ It included 14 studies of AS and two studies of non-radiographic axial SpA (nr-axSpA), totalling 4753 participants across all studies. Participants were recruited mainly from hospital settings such as rheumatology clinics, assessing depression using the well-validated Hospital Anxiety and Depression Scale (HADS). In individual studies included in this meta-analysis, the prevalence of depression ranged from 11% to 64% depending on the criteria and threshold used.^31^ According to this meta-analysis, pooled prevalence of mild depressive symptoms in patients with AS, axSpA and nr-axSpA was 38%, and that for moderate/severe depression (defined as HADS score ⩾11) was 15%.^31^ The prevalence of depression was similar between AS, axSpA and nr-axSpA cohorts. Patients with depression had significantly worse SpA disease activity and greater SpA-related functional impairment. Younger age was associated with higher prevalence of depression.^30^ It is unclear why age was inversely correlated with risk of depression; however, it can be speculated that earlier onset rheumatological disease may cause a greater psychological burden due to increased severity and perceived impact on activity/functioning compared with healthy young peers. The majority of included studies recruited participants from hospital settings, which may result in higher SpA disease activity and functional impairment than primary care samples. This could contribute to a higher prevalence of depression in these cohorts. However, the authors state that there was no correlation between disease activity and depression prevalence estimates and suggest other confounders such as smoking and deprivation could account for the higher disease activity and functional impairment in depressed groups.

There are relatively fewer studies of the prevalence of depression in patients with PsA. A recent meta-analysis on this topic included three studies that collected participants from hospital settings such as outpatient facilities, rheumatology and dermatology clinics across South America, Europe, Canada, and Asia-Pacific, including a total of 1141 patients with PsA.^38^ Prevalence of moderate depression, assessed by HADS or the patient health questionnaire-9 (PHQ-9) criteria, across these studies ranged from 9% to 22%, with a pooled prevalence of 15% [95% confidence interval (CI), 9–21%], which is similar to prevalence of moderate depression in AS. However, these studies did not report prevalence for mild mood disorders in PsA. There is evidence that depression and anxiety is greater in PsA compared with psoriasis without arthritis, suggesting that risk for mood disorders could be associated with disease-related factors.^41^ Interestingly, depression has been reported to increase the risk of PsA in people with psoriasis. In a study using 25-years of primary care medical records, 73,447 patients with psoriasis were identified. Diagnosis of major depressive disorder (MDD) was reported to increase the risk of developing PsA by 37%, even when controlling for multiple covariates (hazard ratio 1.37; 95% CI, 1.05–1.80).^42^ In another longitudinal study of PsA, pain was associated with subsequent development of depression and *vice versa*.^43^

With regards to incidence of depression in patients with AS and PsA, two recent systematic literature review and meta-analyses compared incidence of depression in these patient groups with that in healthy populations.^37,44^ Comprising 5947 patients with AS from three studies conducted in Taiwan, Sweden and the United States (US), a pooled rate ratio (RR) for depression in AS was 1.51 (95% CI, 1.28–1.79).^37^ This 51% increase in risk was calculated based on studies that report incidence rates within 1 year,^45^ 10 years,^46^ and 13 years of developing AS.^47^ Smoking and lower education were reported to be associated with a higher risk of depression.^48,49^ The literature search for this review was carried out in 2018, therefore missing more recent studies. One large-scale nationwide study from South Korea based on 11,465 newly diagnosed cases of AS between 2010 and 2014 reported that, compared with age- and sex-matched controls, there was a two-fold increased risk of incident depression in patients with AS, even after controlling for potential confounders and comorbidities (adjusted hazard ratio 2.21; 95% CI, 2.06–2.36).^50^ Moreover, the cumulative incidence of depression grew over a 6-year period, suggesting that risk of depression increases over time in patients with AS.^50^ In this sample, female sex, lower socioeconomic status, and the number of comorbidities were associated with a higher risk of developing depression.^50^ Similarly, a nationwide population-based study from Taiwan examined the risk of depression in 2331 patients with AS during a median 6-year follow-up period. The study reported that following AS diagnosis patients had a three-fold increased risk of depression in the first year and two-fold increased risk after ⩾5 years.^46^

Regarding the incidence of depression in PsA, four studies were included in a meta-analysis.^44^ These studies comprised a total of 28,614 patients with PsA from the US, and assessed annual incidence following PsA diagnosis. The pooled incidence rate ratio for depression was reported to be 1.44 (95% CI, 1.20–1.73), which was independent of PsA severity. The authors observed that, despite increased rates of depression, only 2.4–13.5% of patients with PsA were taking antidepressant medication, suggesting that many PsA patients who have comorbid depression may not be receiving treatment for their depression. Although incidence of depression appears to be elevated in AS and PsA, incidence may be over-estimated in hospital-based samples due to increased surveillance.

## Common risk factors for depression in AS or PsA patients

Depression is a complex multifactorial disorder with contributions from both environmental and genetic factors. Female sex, exposure to stressful life events in childhood/adulthood, and socioeconomic deprivation are some of the most extensively replicated factors associated with depression.^51–54^ Examples of stressful life events reported to be associated with depression include bereavement, physical, emotional or sexual abuse, relationship breakdown, lack of employment or confiding relationship, and financial problems.^52,53^ At the physiological level, effects of life stressors are possibly mediated by activation of the hypothalamic-pituitary-adrenal (HPA) axis and low-grade systemic inflammation.^55–58^ Stressful life events could be a useful marker for identifying those potentially at risk of depression in clinical settings. History of depression is also a significant risk factor for subsequent depression,^59^ which could be another important risk indicator in clinical settings.

The association of stressful life events and deprivation with depression have also been replicated in patients with AS, axSpA, and psoriasis. For instance, stress and lower household income have been reported to double the risk of depression in axSpA.^60^ As mentioned in the previous section, lower socioeconomic status or educational achievement, smoking and number of comorbidities have been reported to be associated with risk of depression in large population studies of AS.^48–50^ A number of comorbidities are common in patients with AS and PsA, including cardiovascular disease,^61,62^ malignancies,^63,64^ gastrointestinal disorders,^65,66^ among others.^64^ Many of these increase the subsequent risk of depression, and/or *vice versa*.^67–69^ Moreover, multimorbidity increases risk of depression dependent on the number of physical morbidities.^70^ Although it is difficult to ascertain whether depression is a result of multimorbidity, AS, or PsA, it is likely multimorbidity increases psychological and immune system stress, and may present potential pathways through which depression and multimorbidity may be linked. In contrast, results regarding association between depression and sex in patients with AS is somewhat mixed; with studies reporting higher risk in females,^50^ males,^71^ or no difference.^47,60^ Therefore, it is possible that the risk of depression in AS could be relatively similar between the sexes. Although there is limited literature on the link between stress, socioeconomic status and PsA, low socioeconomic status has been found to increase the risk of depression [odds ratio (OR) 1.17, 95% CI 1.08 1.26], anxiety (OR 1.11, 95% CI 1.01 1.23) and mixed anxiety/depression (OR 1.32, 95% CI 1.21 1.45) in psoriasis when compared with healthy controls. Furthermore, low socioeconomic status increased the cumulative probability of anxiety and depression between the ages of 40 and 60 years.^72^

## Potential mechanisms of depression in AS or PsA patients

### Role of disease-related factors

Disease severity, poor quality of life and particular symptoms of AS such as fatigue and sleep disturbance have been reported to be associated with depression in patients with AS. In one study, for every unit increase in the Bath AS Disease Activity Index (BASDAI; scale 0–10) the risk of depression in patients with AS increased by 30%.^60^ In another study, although both disease activity (BASDAI) and AS quality of life scores were moderately correlated with depression (*r* = 0.50 and 0.60, respectively), multivariable linear regression showed that only poor quality of life was independently associated with depression after taking into account both scores.^73^ However, disease activity could still be an important risk factor, given the magnitude of its correlation with depression (*r* = 0.50). Although it is apparent there is a relationship between disease activity, quality of life and depression, cross-sectional data such as these are unable to ascertain the direction of association (i.e. whether depression is a cause or consequence of poor quality of life, disease activity). Longitudinal studies are required to address this issue.

Fatigue is a common symptom in AS, with two studies reporting a correlation with depression severity (*r* ⩽ 0.55).^74,75^ Considering these two studies were based on relatively small samples (80 and 65 cases of AS), the correlation estimates are relatively large and may indicate a significant role of fatigue in depression in patients with AS. Similarly, sleep quality appears to be related to depressive symptoms. In samples of 318 and 80 patients with AS, Spearman’s correlation between sleep problems and depression was reported to be 0.47 and 0.31, respectively.^76,77^ Despite moderate correlations between disease related factors and depression, it is difficult to ascertain from cross-sectional data whether fatigue, sleep problems, quality of life and/or disease activity are a cause or consequence of depression. This is because depression could cause or exacerbate these factors of AS, or both depression and AS may share pathophysiologic mechanisms that contribute to the development of these symptoms.

Similarly, disease-related factors can also lead to depression in PsA. Although directionality has not been investigated, PsA disease activity is greater in patients with depression and/or anxiety.^78^ Similar to AS, sleep and fatigue have been reported to be associated with poor quality of life and risk of depression in patients with PsA.^30,79,80^ Other PsA symptoms may further affect depression risk. For instance, psoriasis has been shown to impair body image.^81^ Using hierarchical multiple regression, psoriasis severity and a younger age of onset has been associated with anxiety, while negative body image mediated the relationship between the severity of psoriasis and depression.^82^ Pain could also be a risk factor for depression. In a longitudinal study pain was associated with subsequent development of depression and *vice versa* in patients with PsA.^43^

### Inflammation as potential shared mechanism for both depression and SpA

Several lines of evidence suggest the potential role of systemic inflammation, a hallmark of SpA and other rheumatological conditions, in the pathogenesis of depression. Autoimmune disease and severe infection are associated with increased risk for subsequent depression, with all 30 autoimmune disorders assessed (including AS and psoriasis) increasing risk.^58^ Interferon treatment, a potent inducer of innate immune response, leads to development of depression in up to 40% of patients with hepatitis C virus.^83^ A meta-analysis of cross-sectional studies have consistently reported increased concentrations of C-reactive protein (CRP) and inflammatory cytokines, such as IL-6 and TNF-α in the peripheral blood and cerebrospinal fluid (CSF) of patients with acute depression compared with healthy controls.^84–87^ Population-based longitudinal studies have reported that elevated circulating IL-6 and CRP levels in childhood are associated with the subsequent development or persistence of depressive symptoms in adulthood,^88–90^ suggesting that inflammation could be a cause, rather than a consequence of depression. Mendelian randomisation studies, which use genetic variants regulating levels/activity of a biomarker as proxies to address the issue of confounding,^91^ suggest that IL-6 and CRP could be potentially causally related to depression, rather than these associations being fully attributable to confounding by lifestyle, stress or other factors.^92,93^

Inflammation appears to be clinically relevant as it is associated with poor antidepressant response.^94,95^ Evidence from clinical trials also supports a role of inflammation in depression, with recent studies pointing to key roles for inflammatory cytokines such as IL-6 and TNF-α. In cohorts that include depressed but otherwise healthy participants and participants with depression and comorbid osteoarthritis, non-steroidal anti-inflammatory drugs (NSAIDs) given as adjuncts to antidepressants are associated with greater symptomatic improvements compared with antidepressants alone in randomised controlled trials (RCTs).^96^ More recently, meta-analyses of secondary outcome data from RCTs of cytokine antagonists, including anti-TNF, anti-IL-6/IL-6R, anti-IL-12/23, anti-IL-4/13 and anti-IL-17 drugs in patients with rheumatoid arthritis, inflammatory bowel disease and other chronic inflammatory physical illness have reported that these drugs improve depressive symptoms independently of improvements in physical illness.^97,98^ Based on seven placebo-controlled studies, there was a moderate effect size (0.40; 95% CI 0.22–0.59) of anti-cytokine treatment on depressive symptoms, with the majority of studies investigating anti-IL-6 and anti-TNF treatments.^97^ In a subsequent review, similar effects were observed, demonstrating the largest antidepressant effects in anti-IL-6 and anti-IL12/23 drug classes. After controlling for physical health outcomes, a trend remained for anti-IL-6, while anti-IL-12/23 effects remained significant, with both showing only marginal attenuation.^98^

Currently a number of RCTs are testing the effects of cytokine and/or cytokine receptor antagonists in patients with depression. However, identifying patients most likely to benefit from immunotherapy is likely to be key for the success of future RCTs, as inflammation is unlikely to be relevant for all patients with depression.^99^ A RCT of infliximab for treatment resistant depression demonstrated treatment response to be associated with higher baseline CRP levels.^100^ A proof-of-concept double blind RCT of the anti-IL-6R mAb tocilizumab in patients with depression is underway, with subjects selected based upon evidence of inflammation and inflammation-related symptoms.^101^

Turning to data from patients with AS, the mood improving effects of infliximab is consistent with a potentially shared role of inflammation in pathogenesis of depression, AS and other chronic inflammatory physical illness.^102^ Sickness behaviour, a response to inflammation in autoimmune disease or infection, is characterised by fatigue, sleep problems, anhedonia, reduced appetite, concentration and motivation, which are also typical symptoms of depression.^103^ The depression syndrome consists of heterogeneous symptoms. Data from population-based studies suggest that elevated levels of circulating IL-6 and CRP are associated with particular symptoms of depression, such as fatigue, sleep and appetite disturbance, which are akin to sickness behaviour.^104,105^

Specific biologic pathways involved in the pathologies of depression and SpA show some concurrence. Whilst the pathogenesis of AS, PsA and depression is yet to be fully understood, recent evidence highlights the IL-17/23 pathway as one of the most important cytokine pathways to contribute to AS and PsA.^6,106–108^ Elevated IL-23 impairs T helper cells, which contributes to the overexpression of IL-17, IL-22, IL-1, IL-6 and TNF. Interestingly, there is evidence of impaired T helper function in depression.^109^ IL-6, IL-1β, TNF-α and CRP are also among inflammatory markers shown consistently to be elevated in patients with depression.^97,110,111^ Nuclear factor (NF)-κB is another marker that has been implicated in both AS and depression,^112,113^ as are inflammasomes.^114–116^ Production of these inflammatory proteins may be activated by psychological stress through the hypothalamic pituitary adrenal (HPA) axis. The HPA axis stimulates the release of glucocorticoids and catecholamines, initiating inflammasome assembly,^109^ which in turn leads to the production of IL-1β and other pro-inflammatory cytokines such as IL-18.^117^

**Figure 1.**
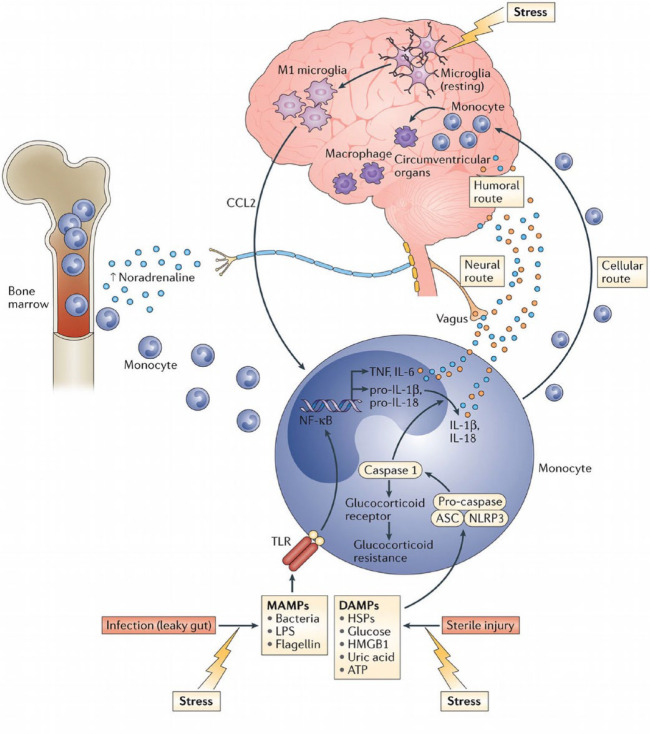
Pathways through which peripheral inflammation can communicate with the brain and influence mood, cognition and behaviour. Cellular route refers to the trafficking and attraction of immune cells to the brain vasculature and parenchyma, such as through the meningeal lymphatic system. Neural pathway involves activation of the vagus nerve by peripheral cytokines, with the signal reaching brain nuclei by retrograde axonal transport. Humoral pathway involves production of cytokines by macrophage-like cells in the circumventricular organs, which can enter the brain by volume diffusion. Figure reproduced from Miller and Raison with permission from Springer Nature.^109^ DAMPs, damage-associated molecular patterns; MAMPs, microbe-associated molecular patterns.

To date, the medical literature has considered the brain as an immune-privileged site, shielded behind the blood-brain barrier. However, current evidence demonstrates a number of pathways through which peripheral inflammation can communicate with the brain. While the exact neurochemical basis of inflammation-induced depression is still being elucidated, current evidence suggests that inflammatory cytokines can influence mood, cognition and behaviour by: (i) decreasing synaptic availability of serotonin by breaking down tryptophan along the kynurenine pathway, and by increasing presynaptic reuptake of serotonin; (ii) increasing oxidative stress due to production of reactive oxygen and nitrogen species; (iii) increasing synaptic glutamate leading to excitotoxicity; and (iv) decreasing neurotrophic support and hippocampal neurogenesis. See Figure 1, and reviews by Dantzer *et al.* and Miller and Raison for a more detailed narrative.^23,109^

### Shared genetic basis for neuropsychiatric and immune disorders

While no data is currently available on a shared genetic basis for depression and AS specifically, emerging evidence indicates a potential shared genetic basis for a number of neuropsychiatric and immunological disorders.^118^ One study investigating genetic variants associated with psychiatric illnesses (schizophrenia, bipolar disorder, major depressive disorder, and autism spectrum disorder) and autoimmune diseases (Crohn’s disease, ulcerative colitis, multiple sclerosis, psoriasis, RA, SLE and insulin-dependent diabetes mellitus) reported evidence for genetic pleiotropy in 24 out of 35 psychiatric-immune disorder pairs.^118^ The strongest pleiotropy was observed for schizophrenia-rheumatoid arthritis with MHC region included in the analysis, and schizophrenia-Crohn’s disease with MHC region excluded.^118^ Using linkage disequilibrium score regression (LDSC) analysis of available genome-wide association studies (GWAS) data, another study reported significant genetic correlations between immune-related disorders and several psychiatric disorders including depression.^119^ RA was the only rheumatologic condition included in this study, which showed evidence for genetic correlations with bipolar disorder, obsessive compulsive disorder, attention deficit-hyperactivity disorder and smoking behaviour. With further GWAS using larger samples, future studies may shed light on potential shared genetic basis for spondyloarthritis, depression and other psychiatric disorders.

## Management of depression in SpA

Depression is a treatable condition with about two-thirds of patients responding to first-line antidepressant treatment.^22^ Depression is associated with poor treatment adherence, poor response to biologics, and higher self-reported disease severity in patients with arthritis.^33,34^ Therefore, it is imperative for clinicians to actively assess and treat depression in AS and other rheumatologic conditions rather than seeing depression as an inevitable by-product of arthritis, pain or poor quality of life. Based on prevalence studies discussed earlier, it is likely that about 40% of patients with AS will have some degree of depressive symptoms while moderate to severe depression is likely to be present in about 15% of patients with AS and PsA.^31,38^ Therefore, routine assessment of depression in rheumatology clinics is necessary to identify these patients, and tailor management strategy accordingly based on depression severity and potential risk.

The UK National Institute for Health and Care Excellence (NICE) recommends depression is assessed in patients with RA on an annual basis. However, it is noteworthy that, with the exception of the National Axial Spondyloarthritis Society (NASS), existing international guidelines for AS do not mention depression, despite this being a common problem in these patients. The NASS recommends that patients with AS see their primary care physician for advice if concerned about their mental wellbeing.

### Assessment of depression and associated risk

The NICE guidelines on the recognition and management of depression in adults with a chronic physical health problem provides clinicians not trained in psychiatry with useful suggestions for assessment of depressive symptoms, severity and potential risks (see Textbox 1 for summary).^120^

**Textbox 1.** Summary of the NICE guidelines for the recognition and management of depression in adults with a chronic physical health problem. Clinical guideline (CG91), October 2009 (Section 1.3).

**Identification and recognition (Section 1.3.1)** 1. Be alert to depression, especially those at greater risk. Consider two questions: ○ during the last month, have you often been bothered by feeling down, depressed or hopeless? ○ during the last month, have you often been bothered by having little interest or pleasure in doing things? 2. If the answer to either is yes and the clinician is trained, three further questions below should be asked. If not trained, a referral should be made to the appropriate specialist and/or general practitioner. ○ during the last month, have you often been bothered by feelings of worthlessness? ○ during the last month, have you often been bothered by poor concentration? ○ during the last month, have you often been bothered by thoughts of death? A practitioner may also want to review patients mental state, associated functional, interpersonal and social difficulties; consider the role of both the chronic physical health problem and prescribed medication in the development or maintenance of depression; and/or ascertain that the optimal treatment for the physical health problem is being provided, seeking specialist advice if necessary. 3. When depression is suspected, consider using a validated measure (for symptoms, functions and/or disability) to inform and evaluate treatment (alternatives provided and suggested for those with communication difficulties). **Risk assessment and monitoring (Section 1.3.2)** 1. If a patient with depression and a chronic physical health problem presents considerable immediate risk to themselves or others, refer them urgently to specialist mental health services. 2. Advise patients with depression and a chronic physical health problem of the potential for increased agitation, anxiety and suicidal ideation in the initial stages of treatment for depression. Patient, family, or carer should be vigilant for mood changes, negativity and hopelessness, and suicidal ideation, and to contact their practitioner if concerned. ○ ensure that the patient knows how to seek help promptly ○ review treatment if patient develops marked and/or prolonged agitation. 3. If a patient with depression and a chronic physical health problem is assessed to be at risk of suicide: ○ take into account toxicity in overdose if an antidepressant is prescribed or the patient is taking other medication; if necessary, limit the amount of drug(s) available ○ consider increasing the level of support, such as more frequent direct or telephone contacts ○ consider referral to specialist mental health services.

The NICE guidelines recommend using a validated tool to assess depression. A number of validated questionnaires for assessing depression are available, such as the PHQ-9, Beck Depression Inventory-II (BDI-II) and HADS.^121^ The PHQ-9 may be particularly useful in rheumatology clinics, as it is a validated, brief, self-report tool widely used to assess depression in clinical and community samples including in UK primary care.^122^ Furthermore, it has been reported that people with arthritis respond to items on the PHQ-9 similarly to those without arthritis, despite the inclusion of somatic items in this scale.^123^ This nine-item questionnaire assesses depressive symptoms occurring in the past 2 weeks. Each item is rated as not present (0), present several days (1), more than half the days (2), and nearly every day (3), giving a total depression symptom score of 0–27. Using established thresholds, the score can provide useful categories of depression: no depression (score 0–4); mild depression (5–9); moderate depression (10–14); moderately severe depression (15–19); and severe depression (20–27). A PHQ-9 score ⩾10 has a sensitivity of 88% and a specificity of 88% for major depression.^122^ The BDI-II could be also useful, as similar to PHQ-9, it focusses mainly on cognitive and psychological aspects of depression, unlike the Hamilton Depression Rating Scale (HAM-D), which includes a number of physical symptoms, making assessment of depression in people with physical illness somewhat tricky. HADS is also used commonly as it measures both depression and anxiety, but it lacks any questions covering suicidal ideation, which is needed to inform risk assessment, an important part of assessment of depression according to the NICE guidelines.

Regarding assessment of suicide risk, it is important for clinicians to know that asking about suicide and related behaviours does not increase the risk of suicide.^124^ Suicidal ideation is common in the general population and in people with depression. According to the British National Psychiatric Morbidity Survey, a general population-based survey conducted in 2000, nearly one in six individuals had had death wishes or considered suicide, while 4.4% of the study population had attempted suicide at some time.^125^ Prevalence of suicidal thoughts in individuals with depression is higher, ranging up to over 60%.^126,127^ Although suicidal ideation is relatively common, certain features may indicate higher risk of suicide attempt, such as having a plan, past suicide attempt, recent onset, and time spent thinking about suicide.^128^ PHQ-9 and some of the other questionnaires mentioned above can be a good starting point for identifying suicidal ideation, followed by further questioning including assessment of aggravating and protective factors, may help clinicians to gauge the need for referral to specialist services.

### Treatment considerations

Assessing severity and risk can inform a stepwise approach to treatment of depression taking into account individual patient preference. According to the NICE guidelines on recognition and management of depression in adults,^129^ antidepressants are not recommended as a first-line treatment in recent onset, mild depression. Rather, active monitoring, individual guided self-help, cognitive behavioural therapy (CBT) or exercise are preferred. According to recommendations from the Canadian Dermatology-Rheumatology Comorbidity Initiative expert group, optimising treatment for rheumatologic condition may also be helpful for depression, as better disease control has been reported to reduce symptoms of depression in patients with RA, PsA, and psoriasis.^130^ Furthermore, a recent systematic review and meta-analysis has reported that anti-cytokine drugs improve depressive symptoms in patients with chronic inflammatory physical illness, such as RA and psoriasis, independently of improving physical illness.^96^

According to the NICE guidelines, antidepressants are recommended for the treatment of moderate or severe depression with SSRIs being recommended as the first-line treatment, prescribed at a dose likely to be effective after titration, if necessary.^129^ Important points to discuss with all patients include choice of drug, utility/availability of non-pharmacological options (e.g. CBT), likely outcome (i.e. gradual relief of symptoms over several weeks), need to continue treatment after resolution of symptoms (e.g. at least 6–9 months after first episode of depression), and risk and nature of discontinuation symptoms.

Although SSRIs are recommended as first-line, use of these drugs in patients with AS and other rheumatologic conditions may be problematic due to increased risk of peptic ulcer and gastrointestinal (GI), uterine, cerebral and perioperative bleeding. A meta-analysis of 42 observational studies reported a significant association between SSRI use and the risk of bleeding (OR = 1.41; 95% CI, 1.27–1.57).^131^ It is thought that SSRIs deplete platelet serotonin leading to reduced ability to form clots and subsequent increase in the risk of bleeding.^131^ Risk factors for bleeding with SSRIs include older age (particularly >65 years), alcohol misuse, coronary artery disease, hypertension, history of GI bleeding, stroke, peptic ulcer, liver disease and medication usage predisposing to bleeding.^132^ Therefore, caution should be exercised when prescribing SSRIs for patients taking NSAIDs, aspirin, anticoagulants or corticosteroids, and for patients with a history of gout, asthma, lupus, psoriasis, etc.^132^

The Maudsley prescribing guidelines in psychiatry, based on limited evidence, suggest that risk of bleeding may be lower with non-SSRI antidepressants that have weak or no effect on serotonin reuptake inhibition, such as mirtazapine, lofepramine and nortriptyline, as opposed to highly potent SSRIs such as fluoxetine or sertraline.^132^ The Maudsley guidelines also suggest that if SSRI use cannot be avoided, to monitor patients closely and prescribe gastro-protective proton pump inhibitors.^132^ Further on drug interaction, SSRIs may increase risk of serotonin syndrome when prescribed with other serotonergic drugs (e.g. tramadol) and electrolyte disturbance on their own and together with diuretics.^132^ Toxicity of medications in overdose may need to be considered especially for patients deemed high risk of overdose/suicide.

### When to consider psychiatric referral

For community-based patients, the NICE guidelines recommend referral to their primary care physician for further assessment when depression is suspected, if the clinician is not trained/competent to perform this assessment (see Textbox 1). Patients should be informed if referrals to specialist mental health services are made. While for the majority of patients a referral to a general practitioner (GP) is likely to be the appropriate route, specialist psychiatric referral should be considered for more complex or high-risk patients. Such scenarios may include treatment resistance (i.e. failure to respond to two or more antidepressants), bipolar depression (antidepressant may induce mania) or multi-morbidity (e.g. comorbid complex/severe physical illness or other psychiatric disorders such as schizophrenia, anxiety disorder, personality disorder, substance use). In such scenarios, specialist psychiatric input could help to inform specific treatment considerations. Risk of suicide may also require more immediate intervention or more specialised assessment and care.

Pathways to specialist mental health care differ widely depending on area or country. For patients in the community, GPs in the UK are best placed to refer patients to appropriate mental health services as they are aware of local service provision and care pathway arrangements. In hospital settings, referrals to specialist liaison psychiatry services would be most appropriate. Liaison psychiatry provides specialist assessment and advice for management of patients across the range of psychiatric conditions, including depression. Naturally, this enables collaboration between specialists in rheumatology and psychiatry, and the best course of treatment can be mutually determined.

## Conclusion

Depression is a major neuropsychiatric disorder, which is common in patients with rheumatological conditions including spondyloarthritis and is associated with poor treatment response and quality of life (see Textbox 2 for summary of key conclusions). Based on meta-analysis, about 40% of patients with AS show some depressive symptoms while moderate/severe depression is present in about 15% of AS and PsA patients. Longitudinal studies indicate that the risk of depression in patients with AS or PsA increases over time. Risk factors for depression in the general population include socioeconomic deprivation and stressful life events such as abuse, relationship breakdown, lack of employment or confiding relationship, and financial problems. In AS and PsA, there are additional disease-related factors that may increase the risk of depression, such as disease activity, quality of life, sleep and fatigue. Furthermore, accumulating evidence suggests a potential causal role for inflammation in depression, which could be a shared mechanism for these conditions.

**Textbox 2.** Summary of key conclusions.

• Better recognition and treatment of depression in rheumatology clinics is necessary as depression is associated with poor clinical outcomes in rheumatology patients. • Patients with SpA have increased risk of depression, with mild depressive symptoms being present in about 40% and moderate/severe depression in about 15%. • SpA-related factors most strongly associated with depression are disease activity, poor quality of life, fatigue, and sleep disturbance. • Emerging evidence indicates that inflammation, particularly proinflammatory cytokines, could be a potential shared mechanism for depression and SpA. • Optimal management of depression in SpA would be aided by routine assessment of depression using validated tools such as the PHQ-9 questionnaire. • Optimising disease control and non-pharmacological interventions such as guided self-help, exercise or psychotherapy is likely to be helpful for mild depression, while antidepressants are recommended for those with moderate/severe depression. • Choice of antidepressant requires careful consideration because of potential risk of adverse effects, such as bleeding, from concurrent use of high potency SSRIs and anti-inflammatory drugs. • Referral to specialist mental health services should be considered for more complex or high-risk patients, such as patients with multimorbidity, antidepressant resistance, substance misuse or suicide risk.

Given the strong associations of depression with poor treatment response, disease severity and quality of life in patients with rheumatologic conditions, it is crucial for clinicians in rheumatology services to actively assess and treat depression. Optimal management of depression in AS would be aided by routine assessment of depression using validated tools such as the PHQ-9 questionnaire. While optimising disease control and non-pharmacological interventions such as guided self-help, exercise or psychotherapy is likely to be helpful for mild depression, treatment with antidepressant drugs is recommended for those with moderate/severe depression. However, choice of antidepressant requires careful consideration because of potential risk of adverse effects, such as bleeding, from concurrent use of high potency SSRIs and anti-inflammatory drugs.

Similar to other common conditions such as hypertension, it is likely that care for the majority of patients with comorbid depression and AS/other rheumatologic conditions will remain under GPs and rheumatology services. Specialist psychiatric referral should be considered for more complex or high-risk patients, such as patients with multimorbidity, antidepressant resistance, substance misuse or suicide risk. In hospital settings, this would involve collaborative working between rheumatologists and liaison psychiatrists to ensure the best possible outcome for patients.
